# How Does Inequality Affect the Residents’ Subjective Well-Being: Inequality of Opportunity and Inequality of Effort

**DOI:** 10.3389/fpsyg.2022.843854

**Published:** 2022-04-06

**Authors:** Qizhi He, Hao Tong, Jia-Bao Liu

**Affiliations:** ^1^School of Statistics and Mathematics, Zhejiang Gongshang University, Hangzhou, China; ^2^Collaborative Innovation Center for Statistical Data Engineering Technology and Application, Zhejiang Gongshang University, Hangzhou, China; ^3^School of Economics and Management, Huaibei Normal University, Huaibei, China; ^4^School of Mathematics and Physics, Anhui Jianzhu University, Hefei, China

**Keywords:** subjective well-being, inequality, opportunity, effort, China

## Abstract

Based on the Chinese General Social Survey database (2010–2015), this article explores the relationship between income inequality and residents’ subjective well-being from the perspective of inequality of opportunity and inequality of effort. We find that inequality of opportunity has a negative impact on subjective well-being in China, where inequality of effort has a positive impact. Our empirical results are robust for changing the inequality indicators. In the sub-sample studies, consistent conclusions are obtained in rural areas, whereas in urban areas only inequality of effort has a significant impact. The results of mechanism study show that inequality of opportunity decreases residents’ sense of fairness, and inequality of effort increases residents’ sense of fairness, thus affecting their subjective well-being. The results of this study provide a good response to the inconclusive research findings on the impact of income inequality on subjective well-being.

## Introduction

The ultimate goal of economic development in all countries is to improve residents’ living standards and well-being. Earlier studies found that a country’s higher gross domestic product does not necessarily mean its residents are happier (Easterlin, 1974). This particular economic phenomenon is known as the “Easterlin paradox” in academic circles. Since Richard Easterlin’s prominent work, many researchers have examined the “Easterlin Paradox” in some high income countries (Clark et al., 2008; Starkauskienë and Galinskaitë, 2015; Antolini and Simonetti, 2019). These studies confirm an apparent contradiction in the data about whether happiness is a function of income. As showed by the Easterlin paradox, based on inter-individual and inter-nation (cross-sectional) income data, there is evidence that happiness is a function of income. But based on intra-individual (time series) comparisons over time spans of more than a decade, income is found to be ultimately unrelated to happiness (Khalil, 2019).

In addition to focusing on the relationship between income and happiness, many researchers try to explain the happiness-income paradox from the perspective of income inequality. But the research results are still inconclusive. A number of studies found that the income inequality has the negative impact on subjective well-being. Using aggregate data from all rounds of the European and the World Values Surveys carried out between 1981 and 2004, Verme (2011) found that income inequality is negatively correlated with life satisfaction. Employing data from the European Quality of Life Survey (EQLS), Delhey and Dragolov (2014) believed that inequality can lead to distrust and status anxiety, which lowers the European’s subjective well-being (SWB). Utilizing European Social Survey data from 29 countries, Hajdu and Hajdu (2014) similarly discovered that Europeans’ subjective well-being increased as income inequality declined. Using two longitudinal data sets from 34 countries, Oishi et al. (2011) documented that income inequality is one of the reasons why subjective well-being does not increase with economic growth.

In contrast, some studies indicated that the income inequality has the positive impact on happiness. Clark (2003) found a significant positive correlation between subjective well-being and income inequality in the reference group. It seems to the respondents that income inequality implies opportunity in some way. Haller and Hadler (2006) found in the study that the inequality of Latin American countries was at high level among all countries in their samples, the residents’ happiness in these countries was also at high level. Other studies showed a non-linear relationship between income inequality and happiness. Employing data from the China General Social Survey (CGSS), Wang et al. (2015) documented that there is an inverted-U shaped relationship between the income inequality and residents’ happiness. Their empirical results are also supported by urban and rural sub-samples. Tavor et al. (2018) divided the Gini values into different ranges and found that at the extreme value of inequality measured by the Gini index, the effect of happiness is negative, while in the middle, the effect of index changes on happiness is ambiguous. Using data from CGSS, Ding et al. (2021) also found that there is an inverted-U shaped relationship between income inequality and subjective well-being for the urban residents.

The differences in research method, or in the selected samples may explain the differences in the relationship between the income inequality and subjective well-being described above. Actually, income gaps just reflect the inequality outcomes, which are associated with the residents’ happiness. It does not mean the existence of income gaps between individuals is unreasonable. Inequality of opportunity may be the most important factor affecting the subjective well-being (He and Pan, 2011). With respect to the egalitarian philosophers, the distribution of justice does not mean the equality of individual outcomes, but requires that individuals own the equal opportunities to achieve valuable results. Equality of opportunity is the best explanation for equality as a distributive ideal (Arneson, 1989).

According to Roemer’s research framework (1993, 1998), the sources of income inequality are mainly composed of two aspects: one is the circumstance factors that individuals can not control, such as, race, gender, family background and so on. Another is the effort factors that individuals can control, such as education and work. The former is called inequality of opportunity and the latter is inequality of effort. Although the issue of inequality of opportunity has drawn wide concern. The related researches mainly focus on the measurement of inequality of opportunity (Bourguignon et al., 2003, 2007; Ferreira and Gignoux, 2011; Bosmans and Öztürk, 2021). There are few papers of which we are aware that study the impact of inequality of opportunity on subjective well-being. He and Pan (2011) constructed the “inequality of opportunity perception index” through three questions related to income equality, educational opportunity and socioeconomic status in the questionnaire, and studied its impact on subjective well-being. As a supplement to the study on this relationship, this article measures the indicators of inequality of opportunity based on the individuals’ annual income, and explores the impact of inequality of opportunity and inequality of effort on residents’ subjective well-being.

The main contributions of this work can be denoted as follows: firstly, we measure the inequality of opportunity index and the inequality of effort index in prefecture-level cities (usually in term of years or countries), which enrich the study of inequality in China. Secondly, we use the measured inequality of opportunity index and inequality of effort index to explore their influence on residents’ subjective well-being, respectively, providing beneficial help for us to understand how inequality affects subjective well-being. Thirdly, we further examine the impact results at provincial level and in urban and rural areas, which support the robustness and heterogeneity analysis of this article. The remainder of this article is organized as follows: Section 2 explains the influence mechanism of inequality of opportunity and inequality of effort on subjective well-being, respectively. Section 3 is the empirical framework. Section 4 describes the data sources and processing. Results are given in Section 5. The influence mechanism test and the robustness test are reported in sections 6 and 7, respectively. The last section is conclusion.

## The Influencing Mechanism of Inequality on Subjective Well-Being

The rise in income inequality does not always have negative effects on society. A degree of inequality can contribute to an economy’s early economic growth by promoting the accumulation of physical capital (Galor and Moav, 2004). The existing studies have shown that inequality has different effects on residents’ subjective well-being in a specific economic system or in different periods. However, previous studies on how income inequality affects residents’ subjective well-being only focus on the inequality outcome, ignoring the “structure” of inequality. According to the introduction part, we believe that these two types of inequality may have significantly different effects on residents’ subjective well-being.

No society can dictate a perfectly equal distribution model in which every individual receives the same absolute amount of income, even in a planned economy. This “unequal” distribution should be considered fair if it is determined by the factors under individuals’ control (Cappelen et al., 2014). That is to say, income gaps caused by the differences in individual efforts are acceptable. But income gaps caused by the external circumstance factors that beyond the individual control are not acceptable. In the latter case, when residents’ income is closely related to factors of individual identity rather than their efforts, equal opportunities may be undermined. Inequality of opportunity reduces the correlation between individuals’ efforts and returns. So it brings in less income mobility, causing a “lock-in effect” on residents’ income expectations (He and Pan, 2011). In addition, whether a person has a local urban hukou (China’s household registration system) is closely related to whether he or she is subject to identity discrimination and discriminated against in a range of policies such as social security and public services (Lu, 2011). Family resources and regional factors are related to educational level, and affect the equality of education opportunity (Wu, 2007). Inequality of opportunities in adult health is influenced by the household socioeconomic status during childhood and the parental education attainment (Fajardo-Gonzalez, 2016). Individuals have an innate aversion to the sense of unfairness. Such identity-related inequalities are inherently unfair and reduce individuals’ happiness (Lu et al., 2014).

Social identities are not just a list of sociodemographic groups that are used to classify individuals (e.g., gender, age, ethnicity, religion). Social identities are relative, and different individuals perceive them as psychological descriptions of themselves (Haslam et al., 2009). They satisfy basic psychological needs such as belonging, self-esteem, control, and meaningful existence (Greenaway et al., 2016). When people lack access to social resources related to education, health, food, housing and mobility, they realize that there is a strong connection between different socioeconomic backgrounds and access to social resources to achieve their goals. Perceived economic inequality makes them highly sensitive to the relevance of cultural capital in sharpening differences between individuals of different status (García-Sánchez et al., 2018). Socioeconomic status comparison becomes a dimension people care about as economic inequality increases. Economic inequality makes people compete with each other and strive for favorable position on the bases of material resources they possess (Melita et al., 2021). Because relative economic status is closely related to residents’ life satisfaction (Cheung and Lucas, 2016). According to status anxiety theory, if a person lives in a society with a large income gap, he may feel great pressure to obtain equality or more social resources than others. As a result, residents’ subjective well-being is negatively affected.

Why a certain degree of income disparity is reasonable and likely to increase residents’ subjective well-being. At the beginning of Chinese reform and opening up, some people were encouraged to get rich first through hard work and legitimate business, and then some people who got rich first would in turn help poor areas and people to become rich. In the process of the economy being allowed to grow in this way, the income gap of residents is gradually widening in China. Individuals show a certain “tolerance” to such income inequality. Residents of poor areas are beginning to imitate some of those who got rich first and move to the cities to find jobs, hoping to increase their family incomes. It’s like driving through a two-lane tunnel, with both lanes heading in the same direction, and getting stuck in a terrible traffic jam, which can be very frustrating. After a while, the cars in the side lane begin to move. Naturally his frustration would be relieved when he knows the blockage has been broken. Although he is still not moving, he will feel better than before, because he has anticipation of his upcoming movement. This visual analogy of wealth can be called “tunnel effect” (Hirschman and Rothschild, 1973). Poor people can also expect their incomes to increase if they think they can do so by increasing their efforts. Such income inequality among residents produces a good demonstration effect, which can make low-income people have expectations for future progress (Knight et al., 2009). In other words, the income gap caused by inequality of effort is tolerable, which makes them have good expectations for the future income increase and believes that efforts can be made to reduce the income gap, thus increasing their subjective well-being.

## Empirical Framework

### The Construction of Inequality Indexes

The inequality of opportunity index and inequality of effort index are the key independent variables of this article. Existing studies on inequality of opportunity provide an important reference for this article. Roemer (1993, 1998) summarizes and develops the theory of inequality of opportunity in a series of studies, establishing a research framework that clearly distinguishes between circumstance and effort variables. The inequality caused by the former is called inequality of opportunity, and the inequality caused by the latter is inequality of effort. On this basis, many scholars have measured the inequality of opportunity. The measuring methods include parametric estimates (Bourguignon et al., 2003, 2007; Carpantier and Sapata, 2013; Gong et al., 2017) and non-parametric estimates (Checchi and Peragine, 2010; Ferreira and Gignoux, 2011; Jiang et al., 2014). Using parametric method to measure inequality of opportunity needs to set up the model in advance, and the measurement results depend on the determined model form. To avoid the influence of model setting error on the measurement results, this article follows Ferreira and Gignoux (2011) and employs the non-parametric method to measure the inequality of opportunity. The framework of measurement of inequality of opportunity and inequality of effort can be defined as:


(1) $$y_{i}=f\,\left(C_{i},E_{i},\mu_{i}\right)$$

(2) $$E_{i}=\left(C_{i},\nu_{i}\right)$$

Where *C*_*i*_ and *E*_*i*_ denote a vector of circumstance (opportunities set) and effort variables, respectively, *y*_*i*_denotes the individuals’ income (advantage). The income variable *y*_*i*_ is influenced by circumstance variables *C*_*i*_, effort variables *E*_*i*_ and random error μ_*i*_. Also effort variables *E*_*i*_ are influenced by circumstance variables *C*_*i*_ and random error ν_*i*_. Supposing that individuals with a total population of *N*, and can be divided into *M* groups according to circumstances variables *C*. Individuals in each group have the same circumstance, that is *C*_*i*_ = *Cm*, *i* = 1,…,*N*, *m* = 1,…,*M*. For individual residents *i* in Group *m*, the individuals’ income can be defined as $\left\{y_{i}^{m}|C_{i}=C^{m}\right\}$, and the distribution function is *F*(*y*,*Cm*). Equality of opportunity implies that circumstance variables have no effect on individuals’ income, and we can denote it as *F*(*y*,*Cm*) = *F*(*y*,*Ck*). Therefore, we can measure inequality of opportunity by comparing the difference of distribution function among different types.

Although the method of stochastic dominance (Lefranc et al., 2009) can help us to determine which type of income distribution function is better, it is difficult to directly apply it in the actual measurement process. Because this method needs a large sample size. In order to better decompose and measure the inequality of opportunity. Ferreira and Gignoux (2011) construct a smoothed distribution $\left\{u_{i}^{m}\right\}$, replacing individuals’ income *y*_*i*_ by calculating the group-specific mean μ*^m^*, to eliminate all within-group inequality. Inequality of opportunity can be denoted as $I\,\left(\left\{\mu_{i}^{m}\right\}\right)$. Further, specific inequality indexes *I*(∙) are expressed in the following Generalized Entropy Index:


(3) $$\theta_{i}=\frac{1}{N}\,\sum_{i=1}^{N}\ln\left(\frac{\bar{y}}{\mu^{m}}\right)$$

(4) $$\theta_{e}=\frac{1}{N}\,\sum_{i=1}^{N}\ln\left(\frac{\mu^{m}}{y_{i}}\right)$$

Where θ_*i*_ denotes the inequality of opportunity index, and θ_*e*_ denotes the inequality of effort index.

### Methodology

The dependent variable in this article, subjective well-being, is an ordered variable of five categories. We use the Ordered Probit model to empirically test the effect of inequality on residents’ subjective well-being. The model is defined as follows:


(5) $$H\,a\,p\,p\,y_{i\,z}^{*}=\alpha_{1}\,O\,I_{i\,z}+\alpha_{2}\,E\,I_{i\,z}+X_{i\,z}^{\prime }\,B+e_{i\,z}$$

(6) $$H\,a\,p\,p\,y_{i\,z}=\left\{ \begin{array}{c} 1,H\,a\,p\,p\,y^{*}\leq a_{0}\\ 2,a_{0}<H\,a\,p\,p\,y^{*}\leq a_{1}\\ 3,a_{1}<H\,a\,p\,p\,y^{*}\leq a_{2}\\ 4,a_{2}<H\,a\,p\,p\,y^{*}\leq a_{3}\\ 5,a_{3}<H\,a\,p\,p\,y^{*} \end{array}\right.$$

Where ***Happy*****_*iz*_** denotes the dependent variable subjective well-being, the subscript *i* and *z* represent the individual residents and the regions (prefecture-level city), respectively. $H\,a\,p\,p\,y_{i\,z}^{*}$ denotes an non-observable latent variable. *OI*_*iz*_,*EI*_*iz*_ and *B* denote opportunity inequality variables, effort inequality variables and other control variables, respectively. a,j(j=0,1,2,3,4) is called the “cutoff point” and is the parameter to be estimated. α_1_,α_2_ and X denote the coefficients of the relevant variables to be estimated.

Supposing that the residuals *e*_*iz*_ follow a standard normal distribution: *e*_*iz*_∼*N*(0,1), and the likelihood function of the sample is as follows:


$$P\left(Happy_{i\,z}=k|OI,EI,B\right)=P\left(a_{k-1}<Happy_{i\,z}^{*}\leq a_{k}|OI,EI,B\right)$$

$$=P\left(Happy_{i\,z}^{*}\leq a_{k}|OI,EI,B\right)-P\left(Happy_{i\,z}^{*}<a_{k-1}|OI,EI,B\right)$$

$$=\phi \,\left[a_{k}-\left(\alpha_{1}\,O\,I_{i\,z}+\alpha_{2}\,E\,I_{i\,z}+X_{i\,z}^{\prime }\,B\right)\right]$$

$$\;-\phi \,\left[a_{k-1}-\left(\alpha_{1}\,O\,I_{i\,z}+\alpha_{2}\,E\,I_{i\,z}+X_{i\,z}^{\prime }\,B\right)\right]$$

Where ϕ(∙) denotes the distribution function of the residuals, we use the maximum likelihood method (MLE) to estimate the parameters.

## Variables Definition and Statistical Description

### Data Processing

This article utilizes Chinese General Social Survey (CGSS) data. The CGSS is a comprehensive and continuous academic survey project on China, conducted by the China Survey and Data Center of Renmin University of China. The empirical study needs to decompose income inequality into inequality of opportunity and inequality of effort at the regional level (prefecture-level city), the sample size has to be large. So in order to solve this problem, we use the survey data from 2010 to 2015 (excluding the data of 2014^1^). We take the CGSS2015 data as a base period and then aggregate data from other years into this year. In the process of aggregating, we have made adjustments in two aspects: Firstly, the annual household income in other years is adjusted according to the CPI of that year and the CPI of 2015. Secondly, the age of individuals from other years is also adjusted to 2015. The total number of aggregated cross-section data is 51574.

To meet the research needs of this article, we processed the data further: (1) Delete the sample with negative annual household income; (2) The answers (including do not know, do not apply, refuse to answer, and do not know clearly) to the relevant questions in the sample are deleted; (3) Delete missing data and other incorrect data from the sample. And the total number of processed data is 42308, of which the sample size in 2015 is 8761, the sample size in 2013 is 9567, the sample size in 2012 is 9947, the sample size in 2011 is 4619, and the sample size in 2010 is 9414.

### Dependent Variable: Subjective Well-Being

The subjective well-being data come from the CGSS database. The question about dependent variable, subjective well-being, in the CGSS questionnaire is “In general, do you think you are happy with your life?” And the answers are: Very Unhappy = 1, Unhappy = 2, Normal = 3, Happy = 4, and Very Happy = 5. Table 1 shows the individual happiness level, mean value and standard deviation from 2010 to 2015.

**TABLE 1:** Statistical description of happiness in China.

Year	Very happy (%)	Happy (%)	Normal (%)	Unhappy (%)	Very unhappy (%)	Mean	Std. Dev.
2010	16.28	56.84	17.28	7.52	2.07	3.7775	0.8795
2011	20.26	60.01	11.17	6.69	1.86	3.9013	0.8602
2012	16.31	60.05	15.11	7.08	1.46	3.8267	0.8358
2013	13.81	59.49	18.16	7.15	1.40	3.7715	0.8243
2015	17.66	60.47	14.42	6.23	1.22	3.8711	0.8145

The average happiness level of residents does not change much and is close to the Happy – level (Happy = 4), other levels of happiness show similar characteristics, which do not change much from 2010 to 2015. For instance in 2015, individuals who report their own happiness accounted for over 60 percent of all respondents, in contrast, fewer individuals reported being unhappy and very unhappy, accounting for 6.23% and 1.22% of all respondents, respectively. Individuals who report being very happy and those who report feeling normal accounted for about 15% of all respondents, respectively.

### Independent Variable: Inequality of Opportunity and Inequality of Effort

In this article, the key explanatory variables are the inequality indexes, including inequality of opportunity and inequality of effort index. There is no precise measure of individual inequality of opportunity and inequality of effort in the questionnaire, and the existing research methods cannot directly measure the opportunity inequality and effort inequality at the individual level. We use the generalized entropy index of the region (prefecture-level city) where the individual residents live as the individual inequality index (He and Pan, 2011), and further decompose it into the inequality of opportunity index and the inequality of effort index.

Firstly, the residents of the same area are grouped into a group according to region where they live (89 groups in total). Then the samples in each group are classified into different types with respect to circumstance variables. The key to measure inequality of opportunity and inequality of effort is how to distinguish circumstance variables from effort variables. There is no consistent division in specific studies. According to inequality of opportunity theory, circumstance variables are defined as all factors that are innate and beyond one’s control. The inequality created by these factors is unacceptable. In the existing representative literature on measuring inequality of opportunity (Bourguignon et al., 2007; Ferreira and Gignoux, 2011), the selection of circumstance variables includes: Race, Father’s and mother’s education, Father’s and mother’s occupation, and region of birth. Efforts is something that individuals can control and take responsibility for, such as working hours, so the resulting income inequality is acceptable. Considering the availability of data and the feasibility of the study, we choose Father’s and mother’s education, Father’s and Mother’s Occupation, Hukou, and Father and Mother’s Party Membership as the circumstance variables. Combined with China’s special urban-rural dual economic structure, no matter what specific variable is chosen, the hukou circumstance variable is the most characteristic and indispensable. In this way, income inequality can be divided into inequality caused by circumstance factors (inequality of opportunity) and the remainder is called inequality of effort.

As the selected circumstance variables increase, different types of residents can be more accurately divided into different groups (Types). While this article measures inequality at a regional level, a potential problem is that some groups may not have sample sizes. So we integrate the Father’s and Mother’s Education, Father’s and Mother’s Occupation, and Father and Mother’s Party Membership into Parental Education (three Groups: below junior high school education, between junior high school education and Senior high school education, over Senior high school education), Parental Occupation (two Groups: full-time job and part-time job), and Parental Party Membership (two Groups: Party Membership and Non-party Membership), respectively. Although we try to include as more circumstance variables as possible to make the measurement of the inequality index more accurate, some variables are missed inevitably. The above method provided a narrow range of lower-boundary for inequality of opportunity (Ferreira and Gignoux, 2011). According to the determined circumstance variables, residents in the same region are divided into different types, and then we measure the inequality index of residents by using the method provided in section “The Construction of Inequality Indexes.”

In addition, there are many other factors affecting the residents’ subjective well-being. According to the existing research literature on subjective well-being (Senik, 2004; Brockmann et al., 2009; Zagorski et al., 2014), we also introduce a series of the same related control variables: Gender (male = 1, female = 0), Age (calculated by the date of birth), Education (number of years of education), Party Membership (party membership = 1, non-party membership = 0), Hukou (urban = 1, rural = 0), Married (married = 1, others = 0), Healthy (healthy = 1, others = 0), Rank (family social hierarchy). Considering that the subjective well-being may be affected by economic growth and may change with time, this article also introduces the degree of economic growth as a control (per capital GDP) and controls the time effect. The statistical description of all independent variables is shown in Table 2.

**TABLE 2:** Descriptive statistics for the variables.

Variables	Obs	Mean	Std. Dev.	Min	Max
Happy	42308	3.8206	0.8428	1	5
OI	42308	0.0453	0.0385	−0.0108	0.2390
EI	42308	0.2535	0.1318	−0.0399	0.5689
Gender	42308	0.5029	0.5000	0	1
Age	42308	51.3381	15.9362	18	101
Educ	42308	8.8651	4.7022	0	19
Party	42308	0.1185	0.3232	0	1
Hukou	42308	0.4092	0.4917	0	1
Married	42308	0.9122	0.2830	0	1
Healthy	42308	3.5852	1.1044	1	5
Rank_high	42308	0.0231	0.1505	0	1
Rank_moderate	42308	0.3237	0.4679	0	1
Ln_GDP	42308	10.5716	0.4562	9.4636	11.6497

## Results

### Full Sample Analysis

As the theoretical analysis points out that the expansion of income gaps may reduce the residents’ subjective well-being, but not all income inequality will reduce residents’ subjective well-being. A certain degree of income gaps may increase residents’ subjective well-being (Knight et al., 2009). We further decompose income inequality into inequality of opportunity and inequality of effort, and explore their effects on residents’ subjective well-being, respectively. Table 3 reports the regression results of the ordered probit model. The regression results are denoted in the column (1): inequality of opportunity has a significant negative influence on residents’ subjective well-being, while inequality of effort has a positive influence on residents’ subjective well-being.

**TABLE 3:** Empirical results of inequality affecting residents’ subjective well-being.

	(1)	(2)	(3)	(4)	(5)	(6)

Variable	Oprobit	Happy = 1	Happy = 2	Happy = 3	Happy = 4	Happy = 5
OI	−0.3169** (−2.03)	0.0119** (2.02)	0.0359** (2.03)	0.0483** (2.03)	−0.0199** (−2.02)	−0.0762** (−2.03)
EI	0.3126*** (6.16)	−0.0118*** (−6.02)	−0.0354*** (−6.14)	−0.0477*** (−6.16)	0.0197*** (6.00)	0.0751*** (6.16)
Gender	−0.0997*** (−8.94)	0.0038*** (8.55)	0.0113*** (8.88)	0.0152*** (8.93)	−0.0063*** (−8.48)	−0.0240*** (−8.94)
Age	0.0055*** (12.16)	−0.0002*** (−11.23)	−0.0006*** (−11.99)	−0.0008*** (−12.13)	0.0003*** (11.07)	0.0013*** (12.15)
Educ	0.0156*** (9.57)	−0.0006*** (−9.13)	−0.0018*** (−9.48)	−0.0024*** (−9.54)	0.0010*** (9.02)	0.0037*** (9.56)
Party	0.2246*** (12.32)	−0.0085*** (−11.39)	−0.0254*** (−12.13)	−0.0342*** (−12.28)	0.0141*** (11.11)	0.0540*** (12.33)
Hukou	−0.0331** (−2.50)	0.0012** (2.49)	0.0037** (2.49)	0.0050** (2.50)	−0.0021** (−2.49)	−0.0080** (−2.50)
Married	0.1053*** (5.02)	−0.0040*** (−4.96)	−0.0119*** (−5.01)	−0.0161*** (−5.02)	0.0066*** (4.94)	0.0253*** (5.02)
Healthy	0.1871*** (35.65)	−0.0070*** (−22.86)	−0.0212*** (−31.93)	−0.0285*** (−34.99)	0.0118*** (21.64)	0.0450*** (35.19)
Rank_high	0.2579*** (7.07)	−0.0097*** (−6.86)	−0.0292*** (−7.04)	−0.0393*** (−7.07)	0.0162*** (6.82)	0.0620*** (7.07)
Rank_moderate	0.1213*** (9.89)	−0.0046*** (−9.37)	−0.0137*** (−9.81)	−0.0185*** (−9.88)	0.0076*** (9.28)	0.0292*** (9.89)
Ln_GDP	0.0334** (0.037)	−0.0013** (2.08)	−0.0038** (−2.09)	−0.0051*** (−2.09)	0.0021** (2.08)	0.0080** (2.09)
Time fixed effect	Yes	Yes	Yes	Yes	Yes	Yes
Obs	42307	42307	42307	42307	42307	42307
Pseudo *R*^2^	0.0226	0.0226	0.0226	0.0226	0.0226	0.0226

The z-value is reported in parentheses to the right of the regression coefficient. The superscripts ***, and ** represent p < 1%, and p < 5%, respectively.

The regression results of ordered probit model can only judge the significance and direction of influence of variables. To give a more intuitive explanation of the regression results, Table 3 further provides the marginal effect of independent variables on different levels of happiness, as shown in columns (2–6). The inequality of opportunity index increases by one unit, the probability of residents feeling “very happy” and “happy” decreases by 7.62% and 1.99%, respectively, and the probability of being “normal,” “unhappy,” and “very unhappy” increases by 4.83%, 3.59%, and 1.19%, respectively. The inequality of effort index increases by one unit, the probability of residents feeling “very happy” and “happy” increases by 7.51% and 1.97%, respectively, and the probability of being “normal,” “unhappy,” and “very unhappy” decreases by 4.77%, 3.54%, and 1.18%, respectively.

**FIGURE 1:**
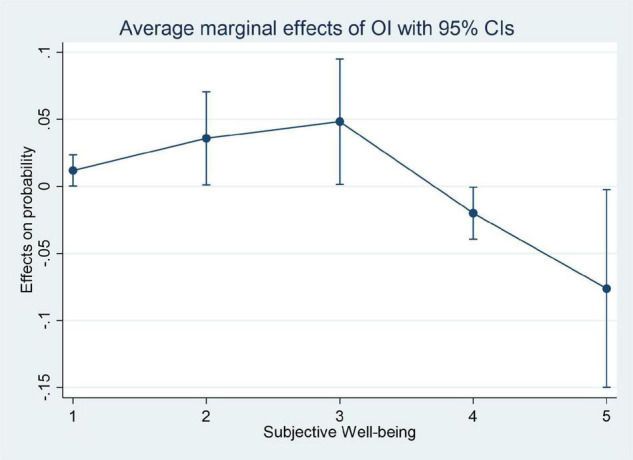
The marginal effect of inequality of opportunity on different subjective well-being.

**FIGURE 2:**
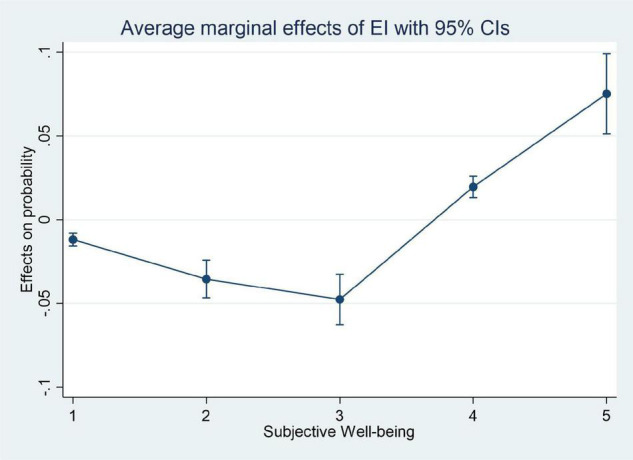
The marginal effect of inequality of effort on different subjective well-being.

Figures 1, 2 clearly show the average impact of inequality of opportunity and inequality of effort on residents’ subjective well-being at different levels, respectively. The directions of influence of the two indexes show opposite results. We can draw the preliminary conclusion that the inequality of opportunity is the main factor that reduces the residents’ subjective well-being, which makes residents feel deprived of equal opportunities or produce a certain degree of anxiety. On the contrary, the inequality of effort will increase the residents’ subjective well-being, which shows the positive “Tunnel Effect” of income gaps.

We also get some important results for other control variables. Considering the limitation of the length of the article, we take column (6) as an example: male reported lower levels of happiness than female, 2.40% percent less on average. It is a common life pattern in Chinese families that men work outside and women take care of the family, men face more social pressure and women are tied down to daily housework that also means a loss of freedom and autonomy, which reduces happiness. On average, social pressure causes more unhappiness than pressure generated within the family. In addition, women are more able to withstand stress and are more likely to be happy than men, which may have something to do with female personality. Residents’ subjective well-being increased by an average of 0.13% with each year of age. The older people are, the more likely they are to achieve career success and enjoy family happiness. Residents’ subjective well-being increased by 0.37% on average for each year of increase in their education level. The more years that residents have received education, the more likely they will get in return and improve their social status, which is beneficial to improve their subjective well-being. Residents with party membership reported an average increase of 5.40% in subjective well-being compared with those without party membership. People with rural hukou are on average 0.80% happier than those with urban hukou. Residents who are married reported an average increase of 2.53% in subjective well-being compared with those who are not married. Residents who are in good health reported an average increase of 4.50% in subjective well-being compared with those with poor health. Residents with higher family rank reported an average increase of 6.20% in subjective well-being compared to those with lower family rank. Additionally, with the increase of economic growth, residents’ subjective well-being will increase.

### Heterogeneity Analysis: Analysis of Urban and Rural Regional Differences

Table 4 demonstrates the empirical regression results of urban and rural areas. Columns (1) and (4) are the regression results of the ordered probit model. Columns (2) and (3) are the marginal effects of inequality of opportunity and inequality of effort on “happy” and “very happy” for urban residents. Columns (4) and (5) are the marginal effects of inequality of opportunity and inequality of effort on “happy” and “very happy” for rural residents. The regression results show that inequality of opportunity and inequality of effort have different effects on subjective well-being among urban and rural residents.

**TABLE 4:** The difference of regional regression results.

Variable	Urban			Rural		
	(1)	(2)	(3)	(4)	(5)	(6)
OI	0.1529 (0.52)	0.0064 (0.52)	0.0380 (0.52)	−0.5039*** (−2.71)	−0.0387*** (−2.69)	−0.1182*** (−2.71)
EI	0.4374*** (4.59)	0.0184*** (4.25)	0.1088*** (4.59)	0.2517*** (4.15)	0.0193*** (4.10)	0.0590*** (4.15)
Control variables	Yes	Yes	Yes	Yes	Yes	Yes
Time fixed effect	Yes	Yes	Yes	Yes	Yes	Yes
Obs	17312	17312	17312	24995	24995	24995
Pseudo *R*^2^	0.0225	0.0225	0.0225	0.0224	0.0224	0.0224

The z-value is reported in parentheses to the right of the regression coefficient. The superscripts *** represent p < 1%, respectively.

The inequality of opportunity has no significant effect on the subjective well-being of urban residents, while the inequality of effort can improve their happiness (0.4374, *z* = 4.59). Perhaps because urban residents generally have a better “circumstance,” where their parents tend to be highly educated, have formal full-time jobs, and come from higher social classes. And the overall difference is not particularly huge for most of the urban residents surveyed. So, urban residents are more likely to perceive the income gaps between them, prompting them to work hard and generate expectations of future income growth, which in turn significantly increases their own happiness.

For rural residents, inequality of opportunity significantly reduces their happiness (−0.5039, *z* = −2.71), and inequality of effort can increase their happiness (0.2517, *z* = 4.15). One possible explanation is that rural residents generally have a worse “circumstance,” and small “circumstance” differences may cause huge income gaps between residents, so the inequality of opportunities caused by the “circumstance” will reduce the subjective well-being of rural residents. Although the direction of influence of core explanatory variables on residents’ subjective well-being is consistent with the regression results of the full sample, the effect intensity is greater in the rural sample, which to some extent reflects the more serious circumstance inequality in rural areas. Similarly, for rural residents, inequality of effort can also increase their subjective well-being through the “tunneling effect.”

### Endogeneity Issue

Although we construct inequality indicators at the regional level (prefecture-city level), endogeneity issue caused by reverse causality is effectively avoided. However, endogeneity caused by missing variables still inevitably exist in the model, which may lead to bias in regression results. We perform CMP estimation methods to address endogeneity between inequality and residents’ subjective well-being. The key to this method is to choose instrument variables (IV) that are highly correlated with inequality variables but not with the random error term.

To be specific, in regard to the geographical location of China, we divide the provinces studied in this article into three regions: the eastern, central and western regions. We use the average value of inequality of opportunity and the average value of inequality of effort of other provinces in the same region as instrumental variables. On the one hand, the instrumental variables selected by us in this way have no direct causal relationship with the residents’ subjective well-being in the province. On the other hand, because the provinces in the same region have similar developmental levels and are closely related to each other, these instrumental variables are highly correlated with the inequality variables (Ge et al., 2021).

Table 5 illustrates the results of endogeneity analysis. Column (1) reports the IV ordered probit regression results. Columns (2–6) report the marginal effect of inequality of opportunity and inequality of effort. After controlling for possible endogeneity issues, the inequality of opportunity significantly reduces residents’ subjective well-being (−23.7128, *z* = −31.96), and the inequality of effort significantly increases residents’ subjective well-being (3.7077, *z* = 22.97), which is consistent with the regression results in Table 3, indicating that the regression results of model are robust.

**TABLE 5:** The regression results with instrument variables.

	(1)	(2)	(3)	(4)	(5)	(6)

Variable	IV oprobit	Happy = 1	Happy = 2	Happy = 3	Happy = 4	Happy = 5
OI	−23.7128*** (−31.96)	4.3482*** (9.08)	1.4884*** (12.12)	1.100*** (8.93)	0.3665*** (6.64)	−7.3031*** (−25.57)
EI	3.7077*** (22.97)	−0.6799*** (−8.38)	−0.2327*** (−12.73)	−0.1720*** (−9.40)	−0.0573** (−6.59)	1.1419*** (19.99)
Control variables	Yes	Yes	Yes	Yes	Yes	Yes
Time fixed effect	Yes	Yes	Yes	Yes	Yes	Yes
Obs	42308	42308	42308	42308	42308	42308

The z-value is reported in parentheses to the right of the regression coefficient. The superscripts ***, and ** represent p < 1%, and p < 5%, respectively.

## Mechanism and Channels Tests

The above empirical study results show that the relationship between inequality of opportunity and residents’ subjective well-being is negative, while the relationship between inequality of effort and residents’ subjective well-being is positive. What are the possible influencing mechanism of inequality variables? Residents’ sense of happiness is a comprehensive category, which is the embodiment of various kinds of pleasure and other emotions produced by individuals in certain social relations. On the one hand, inequality of opportunity creates a sense of unfairness. Such an unfairness circumstance may produce great disparity of individuals’ social status, and the comparison between the individuals will further cause negative feelings such as estrangement and jealousy, leading to the decrease of residents’ subjective well-being. On the other hand, inequality of effort increases the sense of individual fairness, leading residents to believe that the harder they work, the happier they are. Therefore, we use causal steps approach (Baron and Kenny, 1986) to examine whether inequality can affect residents’ subjective well-being through “fairness” channels.

Table 6 reports the results of the impact of inequality on residents’ sense of fairness and subjective well-being. Column (1) shows the effects of inequality of opportunity and inequality of effort on residents’ sense of fairness. The coefficient of inequality of opportunity is significantly negative (−0.4287, *z* = −2.82). The coefficient of inequality of effort is significantly positive (0.7026, *z* = 14.29), indicating that inequality of opportunity can decrease residents’ sense of fairness and inequality of effort can increase the residents’ sense of fairness. Column (2) shows fairness mediating variable has a significant effect on subjective well-being (0.3070, *z* = 57.72). Column (3) shows the results of adding fairness variable to the original regression model. After adding fairness variable, the variable of inequality of opportunity becomes no longer significant, while the variable coefficient magnitude of inequality of effort decreases significantly, indicating that inequality of opportunity has the effect on subjective well-being entirely through the residents’ sense of fairness. And the inequality of effort partly influences the subjective well-being through residents’ sense of fairness.

**TABLE 6:** The results of influencing mechanism.

	(1)	(2)	(3)

Variable	Fair	Happy	Happy
Fair		0.3070*** (57.72)	0.3062*** (57.45)
OI	−0.4287*** (−2.82)		−0.2277 (−1.44)
EI	0.7026*** (14.29)		0.1260** (2.46)
Control variables	Yes	Yes	Yes
Time fixed effect	Yes	Yes	Yes
Obs	42307	42307	42307
Pseudo *R*^2^	0.0167	0.0570	0.0571

The z-value is reported in parentheses to the right of the regression coefficient. The superscripts ***, and ** represent p < 1%, and p < 5%, respectively.

## Robustness Tests

### Change the Circumstance Variables

In order to verify the reliability of the regression results, we replace the circumstance variable of hukou with the circumstance variable of family social rank, and further determine the type of circumstance to which residents belong. We use the method in section “The Construction of Inequality Indexes” again to remeasure the inequality of opportunity index and inequality of effort index of residents, and further test their impact on residents’ subjective well-being. Table 7 shows the empirical regression results of robustness tests. Column (1) is the regression results of the ordered probit model. Columns (2–6) are the marginal effects of inequality of opportunity and inequality of effort on residents’ subjective well-being. No matter the regression results of ordered probit model or marginal effect analysis, the regression results in Table 7 are consistent with those in Table 3. The conclusions of this article are robust when we change the circumstance variable.

**TABLE 7:** Robustness tests: Change the circumstance variable.

	(1)	(2)	(3)	(4)	(5)	(6)

Variable	Oprobit	Happy = 1	Happy = 2	Happy = 3	Happy = 4	Happy = 5
OI	−0.3924** (−2.34)	0.0148** (2.33)	0.0444** (2.34)	0.0598** (2.34)	−0.0247** (−2.33)	−0.0943** (−2.34)
EI	0.3210*** (6.27)	−0.0121*** (−6.12)	−0.0363*** (−6.25)	−0.0489*** (−6.27)	0.0202*** (6.10)	0.0772*** (6.27)
Control variables	Yes	Yes	Yes	Yes	Yes	Yes
Time fixed effect	Yes	Yes	Yes	Yes	Yes	Yes
Obs	42307	42307	42307	42307	42307	42307
Pseudo *R*^2^	0.0226	0.0226	0.0226	0.0226	0.0226	0.0226

The z-value is reported in parentheses to the right of the regression coefficient. The superscripts ***, **, and * represent p < 1%, p < 5%, and p < 10%, respectively.

### Regression Based on Provincial Inequality Indexes

Employing the provincial inequality indexes and the method in section “The Construction of Inequality Indexes,” we empirically test the impact of inequality of opportunity and inequality of effort on residents’ subjective well-being. Table 8 reports the regression results. Column (1) is the regression results of the ordered probit model. Columns (2–6) are the marginal effects of inequality of opportunity and inequality of effort on residents’ subjective well-being. The results we can get from the first column (1) is that inequality of opportunity has a significant negative impact on residents’ subjective well-being (−0.4398, *z* = −1.77), while the inequality of effort has a significant positive impact on residents’ subjective well-being (0.5377, *z* = 7.83). The marginal effects in columns (2–6) are consistent with those in Table 3, except that the significance of inequality of opportunity coefficient decreases, indicating that the empirical regression results are robust.

**TABLE 8:** Robustness tests: Use provincial inequality indexes.

	(1)	(2)	(3)	(4)	(5)	(6)

Variable	Oprobit	Happy = 1	Happy = 2	Happy = 3	Happy = 4	Happy = 5
OI	−0.4398* (−1.77)	0.0165* (1.77)	0.0498* (1.77)	0.0670* (1.77)	−0.0276* (1.77)	−0.1057* (1.77)
EI	0.5377*** (7.83)	−0.0202*** (−7.56)	−0.0608*** (−7.79)	−0.0819*** (−7.83)	0.0338*** (7.52)	0.1292*** (7.83)
Control variables	Yes	Yes	Yes	Yes	Yes	Yes
Time fixed effect	Yes	Yes	Yes	Yes	Yes	Yes
Obs	42307	42307	42307	42307	42307	42307
Pseudo *R*^2^	0.0229	0.0229	0.0229	0.0229	0.0229	0.0229

The z-value is reported in parentheses to the right of the regression coefficient. The superscripts ***, and * represent p < 1%, and p < 10%, respectively.

## Conclusion

Existing papers have conducted a large number of constructive studies on residents’ subjective well-being from the perspective of income inequality by using different methods and data, but have not reached a unified conclusion. Distinguishing between “fair” income inequality and “unfair” income inequality, this article uses China’s data from 2010 to 2015 to decompose income inequality into inequality of opportunity and inequality of effort. We empirically test their impact on residents’ subjective well-being, and attempt to explain the differences in these results from the perspectives of unfairness effect and positive tunneling effect.

It is found that the inequality of opportunity has a significant negative impact on residents’ subjective well-being. The inequality of opportunity caused by circumstance factors will lead residents to have a sense of unfairness or anxiety, which is the reason for the weakening of residents’ subjective well-being. The inequality of effort has a significant positive impact on residents’ subjective well-being, which may make people maintain good expectations for the future income increase and the narrowing of income gaps, thus enhancing their subjective well-being through the “tunneling effect.” Based on a regional heterogeneity study, we find that only the inequality of effort has a significant positive impact on residents’ subjective well-being in urban areas. In rural areas, inequality of opportunities and inequality of efforts both have significant effects on residents’ subjective well-being. In the process of investigating the possible influencing mechanism, we confirm that fairness plays an critical mediating role in the impact of inequality of opportunity and inequality of effort on subjective well-being. This reminds us of the importance of paying attention to and distinguishing between unfair income gaps caused by gender, race, etc., and income gaps caused by differences in individual efforts.

Therefore, while paying close attention to the income gaps of residents, more importantly, we need to create a fair environment for residents and reduce the factors that make residents dislike. As for inequality of effort, it may give a sense that everyone can improve their subjective well-being through hard work. Ideological beliefs related to meritocracy, upward social mobility, attributes about the rich enable hardworking people to have rosy expectations of the future and accept such inequality. In fact, in modern society, resources are increasingly concentrated in the rich and the income gaps between the rich and the poor is increasing, which is one of the severe problems China faces at the present stage. Therefore, accepted such beliefs may create a trap that perpetuates the inequality outcomes and there is intergenerational transmission, further creating inequality of opportunity. In general, while actively maintaining the sound operation of the society, the government should pay more attention to reducing or eliminating inequality of opportunity, forming a reasonable social system and a fair social environment through legislation and other means.

In sum, our discussion shows that it is indeed important to distinguish between inequality of opportunity and inequality of effort while studying the relationship between inequality and subjective well-being. It should be pointed out that since we did the inequality index decomposition at regional level, there may be many interfering factors in the use of regional variables to explain individual behavior in causal inference. And there is a complex interaction between circumstance and effort factors in the process of determining personal income (Gong et al., 2017). Therefore, in the case of quantitative analysis, the impact of inequality of effort on inequality of opportunity needs to be focused. Also the regression results are valid under the implicit assumption that individuals’ self-reported happiness can be compared (Yang et al., 2019), but happiness is a subjective feeling, and thus the value measured by the surveyed data may be influenced by the respondent’s felling at the time. With the rapid development of the information science and computer technology, internet data are crawled and applied in the financial field (He et al., 2021), and crawling the text data having micro characteristics and then constructing the object index of happiness should be carried out in the future.

## Data Availability Statement

The raw data supporting the conclusions of this article will be made available by the authors, without undue reservation.

## Author Contributions

HT performed the material preparation, data collection and analysis, and wrote the first draft of the manuscript. All authors contributed to the study conception and design, commented on previous versions of the manuscript, and read and approved the final manuscript.

## Conflict of Interest

The authors declare that the research was conducted in the absence of any commercial or financial relationships that could be construed as a potential conflict of interest.

## Publisher’s Note

All claims expressed in this article are solely those of the authors and do not necessarily represent those of their affiliated organizations, or those of the publisher, the editors and the reviewers. Any product that may be evaluated in this article, or claim that may be made by its manufacturer, is not guaranteed or endorsed by the publisher.

## Footnotes

1 CGSS2014 is a special survey: Chinese Elderly Society Tracking Survey. At present, the data of 2014 is not released to the public.
